# COVID-19 Open-Data a global-scale spatially granular meta-dataset for *coronavirus* disease

**DOI:** 10.1038/s41597-022-01263-z

**Published:** 2022-04-12

**Authors:** Oscar Wahltinez, Aurora Cheung, Ruth Alcantara, Donny Cheung, Mayank Daswani, Anthony Erlinger, Matt Lee, Pranali Yawalkar, Paula Lê, Ofir Picazo Navarro, Michael P. Brenner, Kevin Murphy

**Affiliations:** 1 Google, Mountain View, California, USA; 2 Department of Artificial Intelligence, Universidad Nacional de Educacion a Distancia (UNED), Madrid, Spain; 3 School of Engineering and Applied Sciences, Harvard University, Cambridge, USA

**Keywords:** Influenza virus, Health policy

## Abstract

This paper introduces the COVID-19 Open Dataset (COD), available at goo.gle/covid-19-open-data. A static copy is of the dataset is also available at 10.6084/m9.figshare.c.5399355. This is a very large “meta-dataset” of COVID-related data, containing epidemiological information, from 22,579 unique locations within 232 different countries and independent territories. For 62 of these countries we have state-level data, and for 23 of these countries we have county-level data. For 15 countries, COD includes cases and deaths stratified by age or sex. COD also contains information on hospitalizations, vaccinations, and other relevant factors such as mobility, non-pharmaceutical interventions and static demographic attributes. Each location is tagged with a unique identifier so that these different types of information can be easily combined. The data is automatically extracted from 121 different authoritative sources, using scalable open source software. This paper describes the format and construction of the dataset, and includes a preliminary statistical analysis of its content, revealing some interesting patterns.

## Abstract (summary)

**Table:** 

Measurement(s)	Epidemiology
Technology Type(s)	Python
Factor Type(s)	cases • deaths • vaccinations
Sample Characteristic - Organism	Homo sapiens
Sample Characteristic - Environment	anthropogenic environment
Sample Characteristic - Location	North America • Central America • South America • Eurasia • Oceania • Antarctica • Africa

## Background & Summary

SARS-CoV-2, the causative agent of the disease known as COVID-19, was identified as a novel coronavirus starting on January 7, 2020 and subsequently declared a pandemic by the World Health Organization (WHO) on March 11, 2020^1^. The health crisis has affected the society and economy of virtually every nation worldwide in ways that might take years to recover from. Although our understanding of COVID-19 and its effects has greatly improved over the last year, there is still much that we do not understand about why the spread of the disease varies so dramatically in different places. Unravelling these effects requires studying the epidemiological outcomes in as many diverse locations as possible, and identifying common patterns. Epidemiological data must be combined with other relevant factors, such as mobility, government interventions, and demographics, in order to try to understand (and control) its spread.

**Fig. 1:**
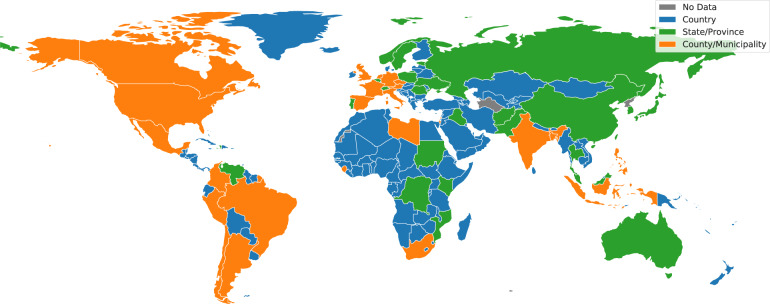
Visualization of the countries for which we have country, state, and county-level epidemiological data. We focus here on data sources that report confirmed cases. We have coverage for 232 different countries or independent territories, from 1,118 different states within these countries (of which, 56 are U.S. states/territories), and 18,478 counties (including all 3,228 U.S. counties).

**Fig. 2:**
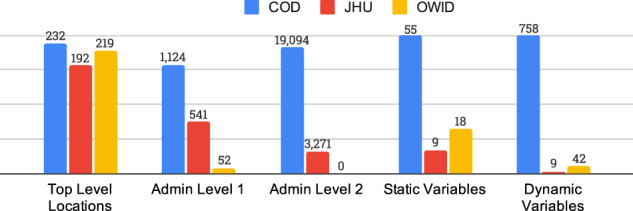
Comparison of our dataset (COD) with Johns Hopkins University (JHU) and Our World In Data (OWID). We plot the number of regions, at administrative levels 0, 1 and 2, for which each data source reports epidemiological data (confirmed cases). We also plot the number of static variables (such as population counts) and dynamic variables (such as mobility) which are available in each repository. While JHU has good data coverage for the U.S., it has significantly less data for international locations, and a minimal amount of non-epidemiological variables. On the other hand, OWID has many more variables than JHU, but the coverage is limited to top level locations and U.S. states. (Note: WorldBank data, which is part of COD, is excluded from this analysis).

In this paper, we introduce the COVID-19 Open Dataset (COD), available at goo.gle/covid-19-open-data. A static copy is of the dataset is also available at figshare^2^. At the time of writing, this is the world’s largest “meta-dataset” (collection of independent datasets) of COVID-related data in terms of number of locations, variables and timespan covered; containing epidemiological information from 22,579 unique locations across 232 different countries and independent territories. For 62 of these countries we have state-level data (admin level 1 regions), and for 23 of these countries we have county-level data (admin level 2 regions). Figure 1 shows a coverage map of our epidemiological data and Fig. 2 compares the coverage with other datasets. Our work aggregates publicly available data, originating from local governments and health authorities (explained in detail in the Data Records section).

**Fig. 3:**
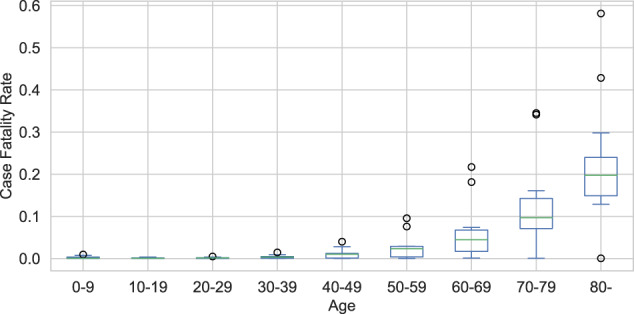
Global case fatality rate for each age group.

**Fig. 4:**
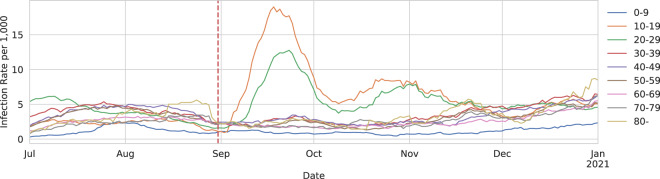
Infection rate for each age group for Alachua county in Florida. The vertical line marks the opening date of the local university.

In addition to indexing outcomes by location, COD also contains outcome data stratified by age or sex. In particular, it contains age stratified data for nearly 48% of all COVID deaths worldwide (870,972 vs 1,801,237, as of the end of 2020). This information can be used to estimate age-dependent case fatality rates across the globe (see Fig. 3), for understanding COVID-19 transmission dynamics in different age groups (see Fig. 4), and so on.

In addition to epidemiological “outcomes” (cases and deaths) mentioned above, COD also contains a lot of data on exogenous “inputs”. A key input is vaccination. As of this writing, we have vaccination data from 186 different countries, 372 states within those countries and 5,928 counties within these states. COD also contains information about various other factors or “covariates” that may affect the spread of COVID. These include information on non-pharmaceutical interventions, human mobility, and static demographic attributes for various regions.

The different data types are stored in 15 different tables, listed in Table 1. All tables use the same set of geographic identifiers (keys), unifying differing standards such as the Nomenclature of Territorial Units for Statistics (NUTS) and the Federal Information Processing Standards (FIPS); this enables the tables to be easily joined and used for various forms of analysis. We give some simple examples of this later in the paper. Whenever possible, we collect data at the county level (admin level 2).

**Table 1:** Summary of the data tables in COD. The “column count” column specifies the number of columns (features) in each data table.

Table	Column Count	Type	Content
index	14	Static	Name and IDs for each location
epidemiology	8	Time	Cases, deaths, tests, recoveries
hospitalizations	9	Time	Hospital data
by-age	150	Time	Epi data stratified by age
by-sex	28	Time	Epi data stratified by sex
vaccinations	6	Time	Vaccination data
lawatlas-emergency-declarations	102	Time	Government mandates
oxford-government-response	20	Time	Government mandates
weather	7	Time	Metereological information
mobility	6	Time	Relative time spent in location types
demographics	18	Static	Current population statistics
economy	3	Static	Current economic indicators
geography	7	Static	Spatial information
health	13	Static	Current health indicators
worldbank	1,404	Static	Latest economic indicators

The “type” column specifies if the data is static (Static), or is a time series (Time).

### Related work

COD contains significantly more data than any other COVID-19 dataset that we are aware of, both in terms of the type of variables and the geographic coverage. In particular, although there are many COVID-19 datasets recording cases and deaths (and in some cases other metrics such as tests, hospitalizations and vaccinations) for the USA — such the New York Times (NYT) dataset https://github.com/nytimes/covid-19-data, the Atlantic’s Covid Tracking project https://covidtracking.com/, and the dataset collected by Bin Yu’s group^3^ — there are very few granular datasets for the entire world.

A notable exception is the CSSE (Center for Systems Science and Engineering) dataset from Johns Hopkins University^4^. This contains country level data for 192 countries, state level data for 23 countries, and county level data for 1 country (the U.S.). However, this is far less coverage than COD (see Fig. 2). In addition, the JHU-CSSE dataset only reports cases and deaths, and does not contain information about hospitalizations, vaccinations, breakdown of cases by age or sex, or other variables, such as government interventions, population structure and health demographics, weather, etc.

Another relevant dataset is Our World In Data. https://ourworldindata.org/coronavirus. However, as we see from Fig. 2, this is limited to country-level epidemiological data (although it does contain a variety of static non-epidemiological variables, such as population size, some of which are incorporated into COD).

Thus we see that COD fills a hole in the data ecosystem, by providing both global coverage at high spatial resolution, as well as broad “semantic” coverage, in the sense of combining multiple kinds of data.

### Applications of COD

Our dataset has already been used in several different research papers^5–7^. It can also be used for vaccine trial planning. When planning trials, it is vital to have granular information about incidence and population demographics, in order to choose a diverse set of suitable sites; our dataset contains the elements necessary for this task.

In the sections below, we provide some preliminary analysis of the data. Since all the code and data is open source, we expect to see more such analyses in the future.

## Methods

In this section, we describe the dataset in more detail, and explain how it was created.

### Data sources

For most locations, there is a single authoritative data source (e.g., a public health agency such as the Centers for Disease Control (CDC) in the U.S.^8^) that provides the data. If this source publishes data in a format that can be automatically processed (for example using a well-established data exchange format such as comma-separated values, as opposed to a data visualization graphic in the form of an image or interactive tool), it is ingested into the data pipeline and considered as the *ground truth*. Such sources correspond to 65% of the data in COD.

For regions where there is no such authoritative data source in a format that can be automatically processed, a journalistic source (e.g., New York Times (NYT)) is used. Failing that, we use crowd-sourcing for manual data extraction (see the Technical Validation section).

Even if a valid authoritative data source exists, sometimes not all of the variables of interest are easily accessible. In this case, a combination of authoritative and journalistic or crowd-sourced data sources are used. For example, U.S. data is published by the CDC, but state-level testing data is not available, so we complement the CDC data source with data from the COVID Tracking Project to capture test counts for U.S. states.

When possible, data for different aggregation levels is ingested separately. For example, U.S. county-level data is collected from the NYT and the CDC datasets, in addition to datasets published by the individual health authorities. Conversely, U.S. state-level data is collected from the NYT and the COVID Tracking project datasets, as well as individual health authorities if they report the data aggregated to the state level.

Even though county-level data could be aggregated into state-level data, many health authorities censor datapoints which could be personally identifiable, for example in very small countries or counties without sufficient cases. An example is the state of Indiana, which suppresses data for counties with less than 10 deaths. Aggregating this data to the state-level would lead to a smaller count than collecting the state-level data directly.

If multiple data sources contain data for the same region and time, then we create a ranked list of sources, and take the values from the most reliable source. Each data source is ranked according to its trustworthiness, reporting cadence and historical reliability.

The reliability is manually estimated by comparing a source with other similar sources. This requires careful analysis of common variables which might be defined differently, such as the time stamp referring to reporting date, collection date, or something else entirely. The initial assumption, which holds true for the majority of data sources, is that data reports are generated at the end of the day and published at the beginning of the following day. We use the timezone of the reporting authority for an initial estimate, and adjust the reported date on a case-by-case basis.

In general, we prefer sources that report historical data rather than values for a single day. When health authorities publish historical epidemiological data, older data can be corrected if an error is found or a reporting methodology is changed.

To compute certain demographic quantities, such as population density, we need to know the size (area) of each location, as well as the total population. Information from OpenStreetMap (https://openstreetmap.org) is used to determine the spatial boundaries of each region, and is processed using Google Earth Engine (https://earthengine.google.com). This is then combined with population information from the WorldPop project (https://worldpop.org) to compute population density.

Details on all the data sources can be found online at the previously mentioned links.

### Creating a unified geo-spatial indexing scheme

All of the data in COD is spatially indexed. This requires a way to define a unique key for each location. Unfortunately, there is no consistent standard across our data sources. For example, some sources use codes from ISO (International Standardization Organization), some use NUTS (Nomenclature of Territorial Units for Statistics), some use FIPS (Federal Information Processing Standards), and some use ad hoc conventions. Thus we had to devise our own hierarchy, and a mapping to this namespace from each source, so we could merge all the data into a unified dataset.

We decided to use a 3 level hierarchy. Level 0 corresponds to the country level. Such locations are identified by ISO 3166-1 codes (e.g., US is the location key for the United States). There are 246 countries in COD. Each country is partitioned into a set of non-overlapping level 1 regions, which (in many countries) correspond to a “state” or “province”. Such locations are identified by an ISO, NUTS, FIPS or locally equivalent ID appended to the country code. There are 1430 level 1 regions in COD, of which 56 are in the USA. (This contains the 50 US states, in addition to Washington D.C. and 5 U.S. territories.) Each level 1 region is further partitioned into a set of non-overlapping level 2 regions, which are often called “counties” or “municipalities”. We have made an effort to acquire as much county level data as possible. In total, there are 20,870 counties in COD, including all 3,225 U.S. counties. The country with the most aggregation level 2 regions is Brazil, with 5,571.

To allow for locations which are not hierarchically nested, we also support aggregation level 3, which we refer to as “localities”. These can be part of one or more locations in aggregation levels 0, 1 or 2. Unlike the other subdivisions, the union of all localities may not correspond to a higher level location in the hierarchy. Localities are also not guaranteed to refer to a distinct geographical location. For example, they could refer to “nursing homes in California”. Most commonly, localities refer to cities, which can be a single aggregation level 2 region, or a combination of several of them — for example, US_NY_NYC is the location key for New York City, which is a combination of 5 aggregation level 2 regions, namely: US_NY_36005, US_NY_36047, US_NY_36061, US_NY_36081 and US_NY_36085. There are 32 localities in COD at the time of writing.

Disambiguating a location can be complicated. For example, there are three different places in Peru named “Lima”: (i) the district of Lima, an administrative level 2 region located within the city of Lima; (ii) the Metropolitan Municipality of Lima, an administrative level 1 region, corresponding to the greater site of Lima; (iii) the province called Lima, which is administrative level 1 and which surrounds the Metropolitan Municipality of Lima but excludes it. Our challenge was to ensure that the data for each region was correctly assigned. Unfortunately, Wikipedia and OpenStreetMap both contain conflicting information. For example, Wikipedia states “Lima Province, which contains the city of Lima, the country’s capital, is located west of the Department of Lima”, yet its reported population count that is given is for the combined Lima Department and Lima Province. Similarly, OpenStreetMap draws the map for Lima Department as the union of Lima Department and Lima Province. When conflicts such as these arose, we resolved them by hand, drawing on extra data sources, such as from Google search.

### Handling age-stratified data

Different sources report age data with different resolutions. To handle this, we specify a mapping from the reported values to 10 different age buckets. Note that some buckets may be empty. For example, a source that reports results for ages 0–18, 19–65, and 65+ would just use the first 3 buckets. In^9^, they present a method to impute the underlying smooth distribution from grouped or binned counts, which could be applied to our data. However, this method makes various assumptions that may be invalid. For example, infection rates are very different for individuals in the age group 10–19 as opposed to 20–29, which can be explained by 18 and 21 being key ages at which people enter different social dynamics. We therefore prefer to keep the raw data, and leave data imputation and analysis to future work.

### System design

In this section, we give a high level overview of the system, which is hosted across GitHub (github.com) and Google Cloud (cloud.google.com).

#### Metadata

The data pipelines use an auxiliary metadata table which contains information about every location known to report data necessary for disambiguation. The information includes labels (using American English names whenever possible) and identifiers which reference other sources of data for a location, such as Wikidata (wikidata.org). Data from any location reported by a data source not found in the metadata table is discarded. Therefore, the auxiliary metadata table represents all locations covered by our repository.

#### Processing the data sources

Individual data sources are encoded as a DataSource object. Each data source goes through the following steps, executed in order: Fetch (download resources into raw data), Parse (convert raw data to intermediate structured format), Merge (associate each record with a known key from the metadata) and Filter (filter out unneeded data and keep only desired output columns).

Additionally, some optional post-processing steps can be executed following the previously described steps, such as: Aggregate (group data by a higher-level location) and Zero-fill (replace null values with zeroes). The majority of the processing in a data source takes place in the *parse* step. All individual records output by the data source have to meet the following criteria:

Each record output by the data source must be matched with a known key present in the auxiliary metadata table, and may include a date column, which must be represented as ISO 8601 format (i.e. YYYY-MM-DD).

Note that we only use non-destructive (invertible) transformations, so we can always recover the original data.

#### Data flow overview

Non-final outputs are saved to increase reproducibility and resiliency. If one step fails to yield a result, the last known good output is used instead. This methodology is crucial in building a reliable service, because data sources can fail to produce valid outputs for a variety of reasons — from transient failures to breaking schema changes. Non-final outputs can either be snapshots of unprocessed data downloaded from the data source or intermediate files, which are the processed outputs from each data source prior to being combined. In addition to increased resiliency, the decoupling of different levels of data processing also provides the ability to perform flexible scheduling for optimal resource allocation.

#### Reliability and monitoring

The reliability and monitoring of the repository are addressed from a data engineering point of view. Each of the components of the data pipelines architecture has extensive unit test coverage, and any errors during processing are automatically logged and reported to an issue tracker. Since errors are expected due to transient issues with data sources, such as server downtime, some of the reported errors are filtered out until they are considered a permanent issue.

Data accuracy is not automatically monitored. This is because it is impossible to distinguish human or processing error from intentional data corrections. For example, on April 24, Spain’s Ministry of Health changed how confirmed cases are counted and started reporting only PCR + results ignoring antibody testing^10^. This led to a decrease in the reported cumulative confirmed counts, which is reflected in our repository. Error reports generated by users are a more accurate form of feedback, but they are limited by the amount of users of the dataset.

## Data Records

Our dataset consists of three main types of data: time series data for biological outcomes of interest, including epidemiological variables (e.g., cases, deaths, hospitalizations) as well as hospitalization-related variables (e.g., number of people in the ICU) and vaccination data; time series data for potentially relevant predictors of these outcomes, such as mobility and government interventions; and static data that describe features of each location that might be relevant (e.g., demographic, economic and health attributes of a population). In total, we aggregated data from 121 different sources. These are stored in 15 different tables, listed in Table 1; more details are also available in appendix A.1 (Appendix), including information on the data sources (which vary by location). We have also created a single aggregated table, by joining 13 of the individual tables. (The aggregated table excludes Lawatlas and Worldbank tables, because they are too wide; however, these are easily joined to the others given the structure of the dataset.) We discuss each of these data sources in more detail below.

The data is available in CSV format at goo.gle/covid-19-open-data. It can also be accessed using Google’s BigQuery cloud database, and a snapshot of the dataset taken at the date of publication is available at figshare^2^.

As part of the presentation of these results, we also demonstrate potential uses of the data by estimating variables of interest such as infection rates compared to vaccination rates and mutual information between specific covariates and epidemiological outcomes. These examples are intended for illustration purposes only, definitive results would require cross-validation with other sources of data and applying domain-specific methodologies.

### Epidemiological data

In this section, we summarize the main types of epidemiological data in COD.

#### Cases, deaths, recoveries and tests

The main epidemiology table has 8 columns (features), corresponding to new and cumulative counts of confirmed cases, deaths, recoveries and tests. See Table A.1.1 (Appendix) for details. Not all of these variables are available at every location; however, there is significant coverage at the state and county level, see Table A.2.1. (Appendix).

We store both new (daily) and cumulative counts. This is because the cumulative counts are not necessarily identical to the sum of daily counts, because many authorities make changes to criteria for counting cases, but do not always make adjustments to the data. In addition, the daily counts can sometimes be negative, due to a correction or an adjustment in the way they were measured. For example, a case might have been incorrectly flagged as recovered on one date so it will be subtracted from the following date.

All the tables in COD use the same set of geographic keys, and hence the data can be easily joined. As an illustration of usefulness of this, we use the population size from the demographics table to compute the death rate per capita at the county level for all the counties in our dataset. Table A.2.2 (Appendix) shows the results. Comparing across such granular geographies reveals patterns that are easily lost when working at the coarser state or country level.

As another example, the external website reproduction.live uses our case and death data to estimate *R(t)*, the effective reproduction number of SARS-CoV-2, for every location in COD. Since it uses exactly the same geographical index, we can easily join their data with ours.

#### Hospitalizations

The hospitalizations table has 9 features, corresponding to new, cumulative and current counts of hospitalized patients, in the ICU, and on a ventilator; see Table A.1.2 (Appendix) for details. We have hospitalization data at the state and county level for 11 different countries; see Table A.2.3 (Appendix) for details.

#### Age-stratified data

For some locations, we have epidemiological and hospitalizations data in age-stratified form (see Table A.1.3 (Appendix) for details). We have this data at state and county level for 18 different countries (see Table A.2.4 (Appendix) for details). Note that different data sources report age data with different resolutions.

As an example for how this data can be used and not intended to be used as definitive calculations, Fig. 3 shows the estimated case fatality rate (CFR; computed as the ratio of reported COVID-19-associated deaths to the total number of reported positive cases) as a function of age. This plot confirms earlier research (e.g.,^11^), which shows that the CFR increases sharply after age 60.

We can also compute the infection rate over time for different age groups, which can reveal interesting trends. For example, in certain university towns, there are different subpopulations of young and older people that have limited social interactions, resulting in different disease dynamics. As an illustration, in Fig. 4, we plot the infection rate over time for each age group in Alachua County, Florida. This is the location of one of the University of Florida campuses. We see a large spike in the infection rate in the 10–19 and 20–29 age groups just after the campus re-opened on 2020-08-31. A similar effect can be seen in Leon County, which is another campus for Florida State University.

#### Sex-stratified data

Some locations also report epidemiological and hospital data stratified by sex. The corresponding by-sex table has 28 columns, corresponding to new and cumulative counts for the following features: cases, deaths, recoveries, tests, hospitalizations, intensive care visits and ventilator usage. See Table A.1.4 (Appendix) for details.

We can use this data to verify the widely reported result (see e.g.,^12^) that males are more likely to die from COVID-19 than females. Using our data, we find that the case fatality rate for males is 3.44%, and for females is 2.69%.

#### Vaccination data

COD contains a growing amount of information about vaccinations. We have data at the state level for countries including the United States, Brazil, Spain, the United Kingdom and Italy, and at the county level for a number of regions including Israel. See Table A.1.5 for details.

**Fig. 5:**
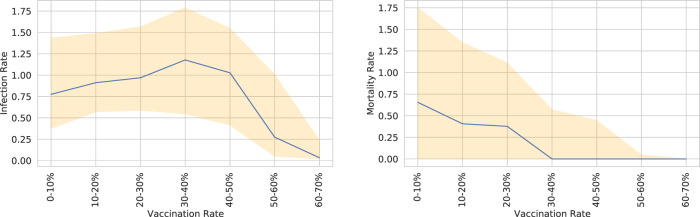
Comparison of vaccination rates and infections (left, per 1,000) deaths (right, per 100,000). Infection and death rates are computed using the latest 30-day window for which the reported vaccination rate range is found. Data excludes locations that have not started vaccination rollout (i.e. vaccination rate is zero). The center line represents the median and the upper and lower bands are the 75th and 25th percentiles.

The effect of vaccinations is very significant once a large enough percentage of the total population is vaccinated. This is illustrated in Fig. 5. We see that infection rates decrease sharply once the majority of the population is vaccinated. We also see that death rates drop rapidly when as little as 30% of the population is vaccinated — this is likely due to the common vaccine rollout strategy, which prioritizes at-risk individuals.

### Static covariates

In this section, we discuss various features or covariates associated with each location that may be useful for forecasting or understanding the spread of COVID-19. The data that we collected is summarized in the tables below. These tables contain statistics that do not change frequently, so the entries do not have a time stamp (but correspond to 2020 values).

- The geography table has 7 features listed in Table A.1.10 (Appendix), including latitude, longitude, elevation, area and OpenStreetMap ID. We have excellent coverage for these quantities at the county level, combining data from Wikipedia with the WorldBank.
- The demographics table has 18 features listed in Table A.1.11 (Appendix). The data in this table combines several different sources, including Wikidata, DataCommons and country specific sources. We were able to construct the data for age demographics by combining population data from WorldPop with region boundaries from OpenStreetMap.
- The economics table has 3 features listed in Table A.1.12 (Appendix), containing various statistics of interest, including gross domestic product (GDP), GDP per person, and the “human capital index”, a metric published by the WorldBank.
- The health table has 13 features listed in Table A.1.13 (Appendix), taken from Wikidata, the WorldBank and Eurostat. There is county level coverage only for life expectancy; we could only find information for the other variables (e.g., smoking or diabetes prevalence) at the country level. Filling this gap is an important topic for future research.
- The World Bank has collected an extraordinary range of data about economic and development indicators from 215 countries. databank.worldbank.org. These range from AG.AGR.TRAC.NO (Agricultural machinery, tractors) to VC.PKP.TOTL.UN (Presence of peace keepers /number of troops, police, and military observers in mandate). In COD, this data is stored in the worldbank table, with 1,404 features.

### Analysis of the static covariates

**Fig. 6:**
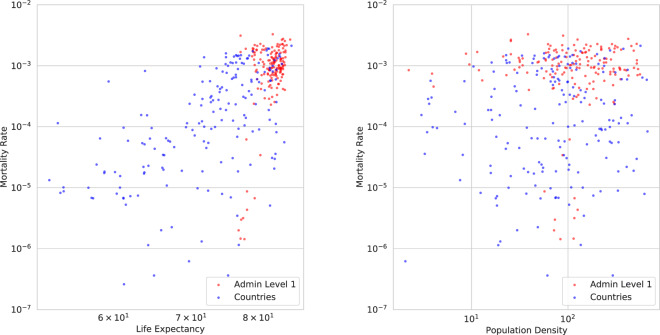
Mortality rates due to COVID-19 versus (left) life expectancy and (right) population density.

We conducted a preliminary analysis of the correlation between some of these static covariates and COVID-19 outcomes, to see if they could explain some of the variation in outcomes. For example, in Fig. 6(a), we plot mortality rates versus life expectancy. We find that the rank correlation coefficient is a modest value of 0.59. This is not surprising, since countries with higher life expectancy tend to have a larger fraction of elderly population compared to other countries, and older people are at higher risk of dying from COVID-19.

Various other factors have been claimed to be predictive of (correlated with) COVID-19 outcomes. For example, in^13^, the authors claim that there is a “moderate association” between population density and infection and mortality rates. However, when using our global dataset of 13,692 locations, we find that neither of these outcomes are correlated with population density. For example, Fig. 6(b), plots mortality rate versus population density. We compute the rank correlation coefficient to be −0.08, indicating no dependence, consistent with some other studies such as^14^.

### Dynamic covariates

In this section, we discuss time series data for features or covariates that might be useful for forecasting or understanding the spread of COVID-19. The data that we collected is summarized below.

#### NPIs

We collected 2 sources of data related to non-pharmaceutical interventions (NPIs). The first one comes from the “Oxford COVID-19 Government Response Tracker” dataset, collected by the Blavatnik School of Government at the University of Oxford^15^. See Table A.1.6 for details. The second datasource is the “Emergency declarations and mitigation policies” dataset collected by the Law Atlas project, which is part of the Policy Surveillance Program at the Temple Center for Public Health Law Research lawatlas.org/datasets/covid-19-emergency-declarations. See Table A.1.7 (Appendix) for details. In the future, we may add other NPI datasets, such as^16^.

#### Weather

In the early days of the pandemic, there was much speculation about whether the virus would be affected by weather, as is the case for the flu^17^. We therefore decided to add weather data to COD. The weather table has 7 features, containing minimum, maximum, and average temperature, rainfall, snowfall, dew point and relative humidity. See Table A.1.8 (Appendix) for details.

#### Mobility

The relative amount of movement of people to potentially crowded places, such as stores and public transit hubs, is a useful leading indicator of COVID-19 spread, as shown in several papers (see e.g.,^18,19^). Fortunately, it is easy to estimate this metric in an aggregated, privacy preserving way using mobile phone data, such as from Google’s “Community Mobility Reports” google.com/covid19/mobility/. See Table A.1.9 (Appendix) for details.

#### Web search

Some studies (e.g.,^20,21^) have shown that internet search results may have some utility as a leading indicator for new COVID-19 outbreaks. We therefore include the “COVID-19 Search Trends symptoms” dataset from Google. This data is available at the country, state and county level for 6 countries (US, Australia, Ireland, New Zealand, Singapore, and the UK).

### Analysis of the dynamic covariates

We conducted a preliminary analysis of the correlation between some of these dynamic covariates and COVID-19 outcomes, to see if they could explain some of the variation in outcomes, and hence be potentially useful for forecasting and furthering our scientific understanding. To avoid biasing our conclusions towards certain modeling assumptions, we adopted a nonparametric approach based on estimating the mutual information (MI) between each covariate time series $\{{X}_{i}(t-L)\}$ and each outcome time series {*Y*_*i*_ (*t*)} for each location *i*, where $L\in \{-14,\ldots ,0\}$ is the optimal lag. To compute MI, we use the widely accepted method described in^22,23^, as implemented in Scikit-Learn^24^. We then visualize the distribution of MI scores across locations *i* for each covariate *X*, and rank the covariates by their median MI.

**Fig. 7:**
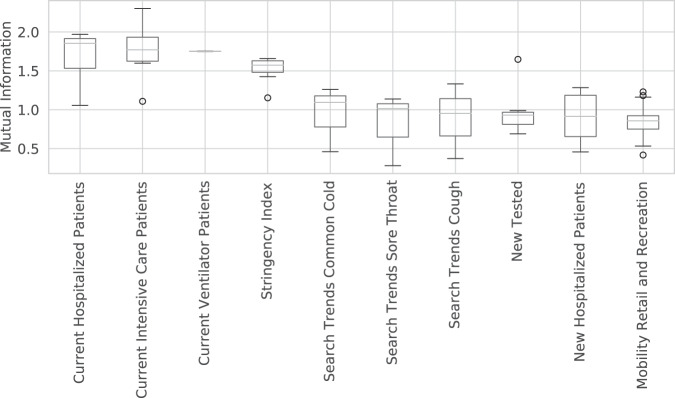
The top 10 time series covariates with highest mutual information with deaths.

We show the top 10 covariates, with highest MI with deaths, in Fig. 7. Again, this analysis simply demonstrates correlation between covariates and epidemiological outcomes. The top two features are the number of patients in the ICU, and the number of patients in hospital, both of which can be explained by well-understood dynamics. The third most important feature is the stringency index, which is an aggregate measure of government intervention. Features 4–6 are search terms related to common COVID-19 symptoms; this seems to suggest that such terms could be a “leading indicator” of COVID-19 outbreaks, as has been reported in other works (e.g.,^25,26^). Features 7–10 are all related to the degree of “social mixing”, as estimated by various signals.

**Fig. 8:**
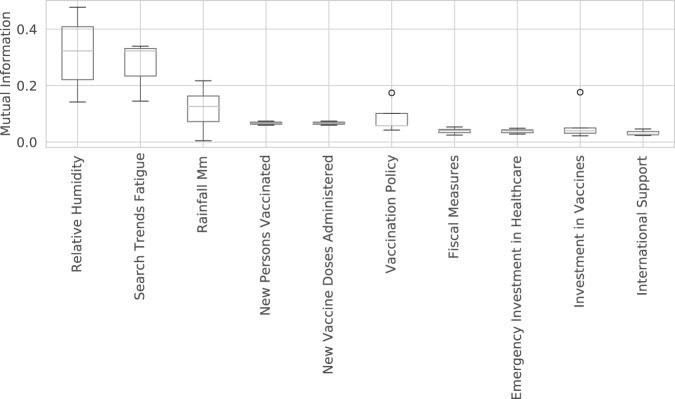
The bottom 10 time series covariates with lowest mutual information with deaths.

The features with the lowest MI (shown in Fig. 8) are consistent with other findings. These include weather-related variables, as well as long-term government policies, which are not related to the current pandemic.

## Technical Validation

Each data source has an associated unit test to ensure that it outputs values for the expected locations using regular expressions. For example, a certain data source may declare that it contains data for U.S. states using the regular expression ^US_[^_] +$. Unit tests enforce that the data source outputs at least one record within the declared location set (U.S. states in this example) and that none of the records match a location that has not been declared.

Data correctness is further validated manually by comparing the data with historically consistent values or, when feasible, by checking the original data source directly. This is done periodically for a subset of the locations covered in our dataset. Automated methods of anomaly detection often produce false positives due to the nature of the data, since health authorities sometimes change how certain variables are measured without always backdating those changes. Because of this, we replicate what the data sources publish as-is and, if deemed necessary, we contact them to seek clarification about specific data points.

Because the infrastructure is fully automated, our repository only contains data sources which can be ingested automatically. This rules out many data sources of interest. For example, historical information about district data from South Africa is only archived by screenshots of annotated maps on social media such as facebook.com/easterncapehealth/photos/a.1752498681685044/2707532132848356. Parsing that information from the unstructured and highly compressed images automatically using computer vision software would likely yield very poor results^27^ and is better done manually.

Evaluating data sources and assessing the reliability of datasets is also a task which requires a significant amount of purely manual work. Sometimes, knowledge of the local language and cultural context is necessary in order to determine the trustworthiness of data sources, or to be able to communicate with the relevant data providers to report issues, clarify how some variables are computed, or request new features in the datasets.

To solve this problem, we collaborated with FinMango (finmango.org), an international nonprofit organization, which used a crowd-sourcing technique to process unstructured data (e.g., screenshots of annotated maps) into structured data. Thanks to this collaboration, we were able to make informed decisions about what data sources to use and how to rank them, and our repository is able to ingest data from multiple regions which do not make historical data available in a format that can be automatically processed by our infrastructure.

## Supplementary information

Supplementary file

## Data Availability

All the code to create the dataset is available at github.com/GoogleCloudPlatform/covid-19-open-data. Jupyter notebooks to reproduce the analyses in this paper are available under the examples folder.
