# Supplementary material for: COVID-19 Open-Data a global-scale spatially granular meta-dataset for coronavirus disease
Source: Sci Data. 2022 Apr 12;9:162. doi: 10.1038/s41597-022-01263-z (PMC9005692; doi:10.1038/s41597-022-01263-z)
Supplement: Supplementary file 1 — Supplementary file [file 41597_2022_1263_MOESM1_ESM.pdf]

A Supplementary material

Contents

References **7**

A Supplementary material **13**

A.1 Details on each data table 14

A.2 Geographic analysis 19

A.1 Details on each data table

Variable	Country	State	County
new/cumulative_confirmed	232	1,124	18,544
new/cumulative_deceased	232	826	17,155
new/cumulative_recovered	19	491	2,869
new/cumulative_tested	114	263	4,310

Table A.1.1. Epidemiology table. We show the coverage for each of the 8 features at each level of spatial granularity. Country-level data from https://www.ecdc.europa.eu/, https://covid19.who.int/ and https://ourworldindata.org/. State and county-level data from country-specific sources. Details at https://github.com/GoogleCloudPlatform/covid-19-open-data/blob/main/docs/table-epidemiology.md.

Variable	Country	State	County
new/current/cumulative_hospitalized_patients	14	259	6,520
new/current/cumulative_intensive_care_patients	6	184	5,982
new/current/cumulative_ventilator_patients	0	16	0

Table A.1.2. Hospital table. We show the coverage for each of the 9 features at each level of spatial granularity. Country-level data from https://ourworldindata.org/. State and county level data from country-specific sources. Details at https://github.com/GoogleCloudPlatform/covid-19-open-data/blob/main/docs/table-hospitalizations.md.

Variable	Country	State	County
new/cumulative_confirmed_age_b	18	359	12,447
new/cumulative_deceased_age_b	11	285	12,138
new/cumulative_recovered_age_b	5	107	7,280
new/cumulative_tested_age_b	4	80	6,185
new/cumulative_hospitalized_patients_age_b	7	155	8,699
new/cumulative_intensive_care_patients_age_b	3	68	5,968
new/cumulative_ventilator_patients_age_b	0	0	0
age_bin_b	-	-	-

Table A.1.3. Age-stratified table. There are 10 age bins, labeled 0 to 9. (Not all locations use all 10 bins.) We show the coverage for each of the 150 features at each level of spatial granularity. Data is from country-specific sources. Details at https://github.com/GoogleCloudPlatform/covid-19-open-data/blob/main/docs/table-by-age.md.

Variable	Country	State	County
new/cumulative_confirmed_sex	16	342	12,850
new/cumulative_deceased_sex	11	242	11,874
new/cumulative_recovered_sex	5	107	7,280
new/cumulative_tested_sex	3	67	6,089
new/cumulative_hospitalized_patients_sex	7	151	8,435
new/cumulative_intensive_care_patients_sex	3	68	5,968
new/cumulative_ventilator_patients_sex	0	0	0

Table A.1.4. Data stratified by sex, which has the values 'male' or 'female'. We show the coverage for each of the 28 features at each level of spatial granularity. Data is from country-specific sources. Details at https://github.com/GoogleCloudPlatform/covid-19-open-data/blob/main/docs/table-by-sex.md.

Variable	Country	State	County
new/current/cumulative_persons_vaccinated	179	342	5,928
new/current/cumulative_persons_fully_vaccinated	116	341	5,928
new/current/cumulative_vaccine_doses_administered	186	372	5,928

Table A.1.5. Vaccination data. We show the coverage for each of the 6 features at each level of spatial granularity. Data is from country-specific sources. Details at https://github.com/GoogleCloudPlatform/covid-19-open-data/blob/main/docs/vaccinations.md.

Name	Type	ID	Description
school_closing	integer [0-3]	C1	Schools are closed
workplace_closing	integer [0-3]	C2	Workplaces are closed
cancel_public_events	integer [0-3]	C3	Public events have been cancelled
restrictions_on_gatherings	integer [0-3]	C4	Gatherings of non-household members are restricted
public_transport_closing	integer [0-3]	C5	Public transport is not operational
stay_at_home_requirements	integer [0-3]	C6	Self-quarantine at home is mandated for everyone
restrictions_on_internal_movement	integer [0-3]	C7	Travel within country is restricted
international_travel_controls	integer [0-3]	C8	International travel is restricted
income_support	integer [USD]	E1	Value of fiscal stimuli, including spending or tax cuts
debt_relief	integer [0-3]	E2	Debt/contract relief for households
fiscal_measures	integer [USD]	E3	Value of fiscal stimuli, including spending or tax cuts
international_support	integer [USD]	E4	Giving international support to other countries
public_information_campaigns	integer [0-2]	H1	Government has launched public information campaigns
testing_policy	integer [0-3]	H2	Country-wide COVID-19 testing policy
contact_tracing	integer [0-2]	H3	Country-wide contact tracing policy
emergency_investment_in_healthcare	integer [USD]	H4	Emergency funding allocated to healthcare
investment_in_vaccines	integer [USD]	H5	Emergency funding allocated to vaccine research
facial_coverings	integer [0-4]	H6	Policies on the use of facial coverings outside the home
vaccination_policy	integer [0-5]	H7	Policies for vaccine delivery for different groups
stringency_index	double [0-100]	-	Overall stringency index

Table A.1.6. Oxford government intervention dataset. This data is available at the country level for 185 countries, and at the state level for U.S., Canada, the U.K. and Brazil. The measures are grouped into three clusters: containment and closure, economic response, and health systems. The stringency index is derived from a weighted combination of C1-C8 and H1. Data is from https://www.bsg.ox.ac.uk/research/research-projects/coronavirus-government-response-tracker. Details at https://github.com/GoogleCloudPlatform/covid-19-open-data/blob/main/docs/table-government-response.md.

Name	Type	Description
lawatlas_mitigation_policy	integer [0-1]	Has the state instituted legal action aimed at mitigation?
lawatlas_state_emergency	integer [0-1]	Is there an emergency declaration in effect in the state?
lawatlas_emerg_statewide	integer [0-1]	Does the emergency declaration apply statewide?
lawatlas_travel_requirement	integer [0-1]	Is there a restriction on travelers?
...	...	...

Table A.1.7. Lawatlas table of government interventions. This data is available for the 50 US states, between the dates 2020-01-20 and 2020-07-01. The data contains a time series of 102 individual measures on a binary 0-1 scale. We only list the first 4 of the 102 features for brevity. Data is from https://lawatlas.org/datasets/covid-19-emergency-declarations. Details at https://github.com/GoogleCloudPlatform/covid-19-open-data/blob/main/docs/table-emergency-declarations.md.

Variable	Country	State	County
average_temperature_celsius	231	1,370	19,486
minimum_temperature_celsius	231	1,370	19,486
maximum_temperature_celsius	231	1,370	19,486
rainfall_mm	231	1,370	19,486
snowfall_mm	73	582	5,758
dew_point	230	1,370	19,485
relative_humidity	230	1,370	19,485

Table A.1.8. Weather table. We show the coverage for each of the 7 features at each level of spatial granularity. Data is from https://www.ncei.noaa.gov/. This data is collected from the Global Summary of the Day (GSOD) published by NOAA (National Oceanic and Atmospheric Association). The values correspond to the average of the ten nearest weather stations within a 300km radius from the centroid of each location. Details at https://github.com/GoogleCloudPlatform/covid-19-open-data/blob/main/docs/table-weather.md.

Variable	Country	State	County
mobility_retail_and_recreation	128	789	4,582
mobility_grocery_and_pharmacy	128	770	4,351
mobility_parks	127	771	3,086
mobility_transit_stations	127	738	2,856
mobility_workplaces	128	833	5,909
mobility_residential	125	733	3,779

Table A.1.9. Mobility reports table. We show the coverage for each of the 6 features at each level of spatial granularity. Data is from https://www.google.com/covid19/mobility/. This contains an aggregated measure of mobility derived from users of Android phones who have opted in to location sharing. In particular, it records the relative increase or decrease of visits to 6 different kinds of locations compared to a baseline (derived from the median day-value from the 5-week period 2010-01-03 to 2020-02-06). This data is available for 128 countries, as well as many states and counties. Details at https://github.com/GoogleCloudPlatform/covid-19-open-data/blob/main/docs/table-mobility.md.

Variable	Country	State	County
openstreetmap_id	235	1,200	13,411
latitude	240	1,382	19,486
longitude	240	1,382	19,486
elevation_m	11	565	14,520
area_sq_km	241	1,329	16,832
area_rural_sq_km	179	0	0
area_urban_sq_km	179	0	0

Table A.1.10. Geography table. We show the coverage for each of the 7 features at each level of spatial granularity. This data is from Wikidata and the World Bank. Details at `https://github.com/GoogleCloudPlatform/covid-19-open-data/blob/main/docs/table-geography.md`.

variable	Country	State	County
population	242	1,246	19,195
population_male	235	1,010	16,294
population_female	235	1,010	16,294
population_rural	213	0	0
population_urban	213	0	0
population_largest_city	152	0	0
population_clustered	121	0	0
population_density	211	240	699
human_development_index	186	39	5,494
population_age_00_09	230	1,010	15,937

Table A.1.11. Demographics table. There are 8 age buckets, labeled 00_09, 10_19, . . ., 70_79 and 80_and_older. We show the coverage for each of the 18 features at each level of spatial granularity. This data is from Wikidata, DataCommons, WorldBank, WorldPop, and country-specific sources. Details at `https://github.com/GoogleCloudPlatform/covid-19-open-data/blob/main/docs/table-demographics.md`.

Variable	Country	State	County
gdp_usd	203	122	8
gdp_per_capita_usd	203	151	9
human_capital_index	156	0	0

Table A.1.12. Economics table. We show the coverage for each of the 3 features at each level of spatial granularity. This data is from Wikidata, DataCommons and the World Bank. Details at `https://github.com/GoogleCloudPlatform/covid-19-open-data/blob/main/docs/table-economy.md`.

Variable	Country	State	County
life_expectancy	205	207	3,025
smoking_prevalence	146	0	0
diabetes_prevalence	209	0	0
infant_mortality_rate	193	0	0
adult_male_mortality_rate	189	0	0
adult_female_mortality_rate	189	0	0
pollution_mortality_rate	183	0	0
comorbidity_mortality_rate	183	0	0
hospital_beds_per_1000	25	0	0
nurses_per_1000	180	0	0
physicians_per_1000	164	0	0
health_expenditure_usd	186	0	0
out_of_pocket_health_expenditure_usd	186	0	0

Table A.1.13. Health table. We show the coverage for each of the 13 features at each level of spatial granularity. This data is from Wikidata, World Bank and Eurostat. Details at `https://github.com/GoogleCloudPlatform/covid-19-open-data/blob/main/docs/table-health.md`.

Country	Number of States	Number of Counties
Afghanistan	34	0
Argentina	24	518
Australia	8	0
Austria	9	94
Bangladesh	8	64
Belgium	11	0
Brazil	27	5570
Canada	13	91
Chile	16	346
China	31	0
Colombia	33	1113
Czech Republic	14	77
Democratic Republic of the Congo	22	0
Estonia	15	0
France	17	96
Germany	16	412
Haiti	10	0
India	34	608
Indonesia	34	507
Iraq	18	0
Israel	7	262
Italy	21	103
Japan	47	0
Kenya	47	0
Libya	13	11
Malaysia	16	0
Mexico	32	2377
Mozambique	11	0
Netherlands	12	355
Norway	11	0
Pakistan	5	0
Peru	26	1798
Philippines	17	111
Poland	16	0
Portugal	7	0
Romania	42	0
Russia	84	0
Sierra Leone	0	16
South Africa	9	52
South Korea	17	0
Spain	19	1045
Sudan	18	0
Sweden	21	0
Switzerland	26	0
Taiwan	9	0
Thailand	75	0
Ukraine	25	0
United Kingdom	13	181
United States of America	55	3223
Venezuela	23	0

Table A.2.1. List of countries for which we have case data at the state and county level.

Location Key	Location Name	Deaths per 100,000
PE_CAL	Peru-Callao	314.0
IT_23	Italy-Aosta Valley	301.6
IT_25	Italy-Lombardy	249.7
CH_TI	Switzerland-Ticino	219.1
ES_CL	Spain-Castilla y León	215.7
US_NJ	United States of America-New Jersey	212.6
PE_ICA	Peru-Ica	210.9
MX_CMX	Mexico-Mexico City	208.2
ES_CM	Spain-Castilla-La Mancha	207.3
ES_AR	Spain-Aragón	198.7
ES_RI	Spain-La Rioja	187.8
AR_C	Argentina-City of Buenos Aires	187.0
IT_42	Italy-Liguria	186.4
MX_BCN	Mexico-Baja California	185.9
PE_LIM	Peru-Lima Region	184.0
US_MA	United States of America-Massachusetts	182.8
CH_NE	Switzerland-Neuchâtel	182.1
IT_21	Italy-Piedmont	181.8
ES_MD	Spain-Comunidad de Madrid	179.7
MX_SON	Mexico-Sonora	178.6
PE_MOQ	Peru-Moquegua	177.3
CH_VS	Switzerland-Valais	176.5
PE_LMA	Peru-Metropolitan Municipality of Lima	176.4
IT_TN	Italy-Trentino-Alto Adige	174.1
IT_45	Italy-Emilia-Romagna	173.5
BR_RR	Brazil-Roraima	173.4
US_SD	United States of America-South Dakota	173.3
CO_91	Colombia-Amazonas	172.1
AR_V	Argentina-Tierra del Fuego	171.4
US_RI	United States of America-Rhode Island	171.3

Table A.2.2. Top 30 subregion level 1 locations around the world with data in COD ranked by death rate per capita.

Country	Number of States	Number of Counties
Argentina	24	506
Belgium	11	0
Brazil	27	5453
Japan	47	0
Mexico	32	0
Netherlands	12	354
Philippines	17	111
Spain	17	0
Switzerland	26	0
United Kingdom	4	14
United States of America	42	72

Table A.2.3. Countries in which we have hospitalization data broken down at the state and county level.

Country	Number of States	Number of Counties
Argentina	24	519
Brazil	27	5570
Colombia	33	1113
Czech Republic	14	77
Estonia	15	0
France	13	96
Germany	16	412
India	34	0
Mexico	32	2377
Peru	26	1850
Philippines	17	111
Spain	17	0
Taiwan	9	0
Thailand	75	0
United States of America	7	356

Table A.2.4. Countries for which we have age-stratified case data at the state and county level.
